# Coprophagia and Entomophagia in a Patient with Alcohol Related Dementia

**DOI:** 10.1155/2017/3109463

**Published:** 2017-08-21

**Authors:** João B. Fonseca, Pedro Morgado

**Affiliations:** ^1^Hospital da Senhora da Oliveira, Guimarães, Portugal; ^2^Hospital de Braga, Braga, Portugal; ^3^Life and Health Sciences Research Institute (ICVS), School of Health Sciences, University of Minho, Braga, Portugal; ^4^ICVS/3B's-PT Government Associate Laboratory, 4710-057 Braga, Portugal

## Abstract

Coprophagia and entomophagia are two phenomena not commonly reported in the medical literature and their occurrence is usually associated with mental disorders. We present the case of a 59-year-old man with a history of alcohol abuse who was evaluated due to cognitive deterioration and disturbed eating habits including feces and living insects. Organic causes were ruled out and an important cognitive impairment became evident on neuropsychological formal test. The behavior remitted after antipsychotic pharmacologic therapy and alcohol detoxification, leaving the diagnostic impression of alcohol related dementia. This report shows a rare association of these two conditions in a patient with dementia.

## 1. Introduction

Coprophagia defines the behavior of consuming one's own fecal waste, and it is usually viewed as a form of pica. Its occurrence has been described in individuals suffering from a wide range of mental disorders such as mental retardation, dementia, schizophrenia, cerebral tumors, obsessive compulsive disorder, depression, alcoholism, and fetishism [1].

There are potential health risks related to coprophagia like salivary gland infection [2] and gastrointestinal disorders caused by parasite infestation [3]. In spite of not being regularly seen in humans, coprophagic behavior has been commonly described among veterinarians and it was suggested that digestive enzymatic deficiencies could provide an explanation for this condition [4].

On the other hand, entomophagia is considered the human consumption of insects for nutritional purposes, and the way these eating practices are accepted is largely variable depending upon the cultural context [5, 6]. The association between entomophagia and psychiatric disorders is not abundant, but at least one case report of this eating practice in a schizophrenic patient has been published [6].

In this report, we describe the case of a patient whose first manifestation of his underlying dementia was coprophagic and entomophagic behavior.

## 2. Case Report

A 59-year-old Caucasian, unmarried, unemployed, elementary school educated male was brought to our hospital for a psychiatric emergency observation. His caregivers indicated that the patient was presenting an abnormal behavior characterized by sexual disinhibition, physical aggression, disturbed eating habits alternating between hyperphagia and food refusal periods, and disconnected speech. The patient's symptoms started approximately 3 years prior to the evaluation. At that time, there was a record of one inpatient psychiatric admission for social disinhibited behavior. Moreover, they reported that the patient had been ingesting his own feces soon after defecation and eating raw small insects that he caught around the living area. There is no history of ingesting other inappropriate objects.

A past medical history was remarkable for the presence of nonmedicated hypertension and diabetes mellitus. Additionally, the patient had abusive alcohol drinking habits that started in the beginning of his adult life, consuming around 50 standard drinks per week for the last 30 years. Excepting his alcoholism, his caregivers referred that the patient was able to work and provided for himself since his youth, having an apparent social expected life until 3 years ago when the cognitive impairments began to show. Since then, his mnesic functions and social functioning had been declining steadily.

The mental status examination during the initial assessment revealed that the patient was vigilant, but disoriented about time and place. The contact with the staff was intrusive. His speech was fluent and perceptible, although sometimes it became disorganized and punctuated with coprolalia. Mood was neutral. He did not present sensoperceptive disturbances or thought content disorders such as delusions. The patient's insight about his clinical situation was absent. On the Mini-Mental State Examination, he scored 21 out of 30.

In order to clarify the diagnosis, the patient was admitted to the psychiatric ward and then submitted to a full neurologic workup including neurologic consultation, brain magnetic resonance imagining, lumbar puncture with biochemical and cytological analysis of the liquor, electroencephalogram, and laboratory testing for viruses (HIV, HBV, and HCV), immunological markers (antinuclear antibodies, rheumatoid factor, and antineuronal antibodies), and vitamins (B12 and folates). Physical examination was normal. None of the exams showed alterations that could be pointed out as an etiologic explanation. The laboratory data showed a negative blood alcohol dosing and normal ammonia levels. Liver enzymes and gamma glutamyl transferase were elevated. Our clinical impression was alcoholic dementia with coprophagia.

A formal neuropsychological test was also applied revealing multidomain deficits regarding simple attention, orientation, remote and short-term memory recalling, and expressive speech. The results were compatible with demential syndrome with a diencephalic (Korsakoff type) and frontal pattern suggestive of alcoholic dementia.

During staying in the hospital, the symptoms ameliorated after introduction of pharmacological therapy with olanzapine 10 mg and alcoholic detoxification. Empirically intramuscular thiamine supplementation was also provided. He was discharged from the hospital after 7 days with adjusted behavior. In the follow-up evaluations (6, 12, 18, and 24 months after discharge), the patient presented adjusted behavior but maintained multidomain cognitive deficits regarding remote and short-term memory recalling and expressive speech.

## 3. Discussion

According to the literature addressing coprophagia phenomena, its occurrence has been mostly found among mentally challenged people.

Although there are not any formal guidelines for the management of coprophagia, several pharmacologic treatments have been attempted with some degree of success, including the use of serotonin reuptake inhibitors, tricyclic antidepressants, antipsychotics, mood stabilizers, dietary supplementation, abstinence from alcohol, and behavioral interventions [1].

We present a case of an individual who had a history of alcohol abuse who was brought to psychiatric attention due to his bizarre and disinhibited behavior which included the ingestion of his own feces and insects. Organic brain causes, like amygdala damage, were ruled out by the request of an MRI. Additionally, he did not present any vitamin deficiencies or any blood count disturbances, particularly iron deficiency or macrocytic anemia, which could be an etiologic factor as pointed out by Sharma et al. [7].

Our patient presented a significant impairment on the psychometric evaluation that showed an important cognitive deficit. This can be an explanation for the eating behavior and comes in line with the conclusion stated by Fairburn and Hope that cognitive impairment is important for the etiology of pica and potentially of coprophagia [8]. Furthermore, it was hypothesized that the disinhibition caused by dementia can lead patients to eat their feces rather than hold or smear them [1]. We know that alcohol related dementia is related to damage to frontal lobes resulting precisely in disinhibition and negligence of the outcomes of one's conduct, including eating practices.

The reports of entomophagia associated with psychiatry disorders are scarce and therefore it is difficult to give a diagnostic meaning to this behavior. However, its significance must be understood taking into account the patient's cultural background in order to integrate that into the symptoms constellation. Eating insets is viewed with revulsion and abhorrence in the Portuguese culture which makes this situation unexpected and bizarre especially when associated with cognitive deterioration.

Behavioral symptoms improved with the introduction of antipsychotics and abstinence from alcohol but cognitive deficits remained, which led us to believe that the global cognitive disorder fitted into alcohol related dementia category.

In this case, we observed an interesting association of two phenomena that are not routinely mentioned in the psychiatric literature and reported that they can also be shown in the course of dementia.

## References

[1] Beck D. A., Frohberg N. R. (2005). Coprophagia in an elderly man: a case report and review of the literature. *International Journal of Psychiatry in Medicine*.

[2] Donnellan C. A., Playfer J. R. (1999). A case of coprophagia presenting with sialadenitis. *Age and Ageing*.

[3] Bugle C., Rubin H. B. (1993). Effects of a nutritional supplement on coprophagia: A study of three cases. *Research in Developmental Disabilities*.

[4] Houpt K. (1982). Ingestive Behavior Problems of Dogs and Cats. *Veterinary Clinics of North America: Small Animal Practice*.

[5] Raubenheimer D., Rothman J. M. (2013). Nutritional ecology of entomophagy in humans and other primates. *Annual Review of Entomology*.

[6] Lingeswaran A., Vijayakumar V., Dinesh J. (2009). Entomophagy and coprophagy in undifferentiated schizophrenia. *Indian Journal of Psychological Medicine*.

[7] Sharma T. R., Aly M., Kavuru B. (2011). Coprophagia and pica in individuals with mild to moderate dementia and mixed (iron deficiency and macrocytic) anemia. *Journal of the American Geriatrics Society*.

[8] Fairburn C. G., Hope R. A. (1988). Changes in eating in dementia. *Neurobiology of Aging*.

